# Phenotypic integration and the organization of discrete facial variation

**DOI:** 10.1111/joa.70176

**Published:** 2026-05-12

**Authors:** Arodi Farrera

**Affiliations:** ^1^ Instituto de Investigaciones Antropológicas Universidad Nacional Autónoma de México Ciudad de México Mexico

**Keywords:** facial features, individualization, morphological integration, morphological modularity, prevalence

## Abstract

Facial morphology results from coordinated developmental and evolutionary processes that produce structured patterns of covariation among traits. Therefore, individual faces reflect integrated anatomical configurations rather than collections of isolated traits. However, discrete facial variation is often examined in a disconnected manner without explicitly accounting for these biological interdependencies. Given that such interdependencies shape which trait combinations are more or less likely, this study evaluates how modularity and integration structure the joint occurrence of facial features. Frontal facial photographs of adult males (*n* = 462) were analyzed. Twenty‐nine anatomical landmarks were digitized and analyzed using geometric morphometrics. Procrustes coordinates were used to test three modularity hypotheses, to quantify integration between facial modules, and to explore patterns of continuous covariation through principal component analysis. Then, morphometric variables were categorized into discrete variables to evaluate. The results confirm that the face is organized into relatively distinct anatomical modules, with varying degrees of covariation. The highest integration observed was between the facial outline and the eyes modules. Observed frequencies of discrete trait combinations further support the structured co‐occurrence of facial features by showing deviations from independence expectation. These findings highlight the importance of interpreting discrete facial phenotypes as integrated systems, providing a framework that complements trait‐based approaches and insights for understanding facial discrete variation.

## Graphical abstract

Integration patterns shape the prevalence of discrete facial trait combinations.

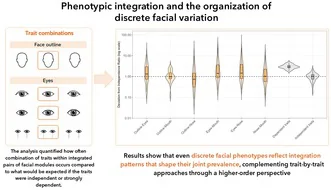

## 1 INTRODUCTION

Human facial morphology is a highly integrated anatomical system shaped by shared developmental pathways, functional demands, and evolutionary processes (Cheverud, 1996; Hallgrímsson et al., 2009; Willmore et al., 2007). At the individual level, these processes influence how traits develop and interact, producing coordinated variation among subsets of anatomical structures (Marcucio et al., 2011), while the cumulative effect of such integrated developmental processes produces population‐level patterns of phenotypic covariation (Hallgrímsson et al., 2009). As a result, some subsets of traits are more tightly connected to each other than to other parts of the phenotype (modularity), with varying degrees of interdependence among them (integration) (Hallgrímsson et al., 2009; Klingenberg, 2014; Zelditch & Goswami, 2021).

From this perspective, individual faces are not random collections of independent traits, but represent a coordinated configuration constrained by shared biological processes (Ferrario et al., 1998; Quinto‐Sánchez et al., 2018; Starbuck et al., 2011). Classic examples, such as the integration between the basicranium and facial morphology, demonstrate how developmental and evolutionary constraints channel phenotypic variation along specific directions of the shape space while limiting variation in others (Klingenberg, 2010), producing relationships between cranial and facial dimensions that are relatively predictable (Bastir & Rosas, 2006, 2016; Lieberman et al., 2000). As traits within highly integrated regions tend to vary together, their joint occurrence is structured rather than random; that is, some trait combinations are common because they align with such a major axis of integrated variation, while others are rare because they require decoupling traits that normally develop together.

This perspective is particularly relevant in contexts where continuous facial variation is operationalized as discrete units used to evaluate how an individual's observed traits compare to population‐level patterns. In the fields of forensic identification, clinical dysmorphology, and biological anthropology, for example, facial variation is often examined on a trait‐by‐trait basis, using standardized approaches that ensure coverage and reproducible procedures across observers and contexts (Ciancia et al., 2024; Fuentes‐Hurtado et al., 2019; Ohlrogge et al., 2009; Vanezis et al., 1996). However, treating discrete traits as independent variables in such contexts overlooks the abovementioned structured covariation and co‐occurrence of facial features.

Building on this perspective, some features, such as nevi or cranial non‐metric traits, are particularly informative for individualizing phenotypes because they are rare and show relatively low covariance with other traits (Black et al., 2014; Cappella et al., 2019; Palamenghi et al., 2023, 2025). In contrast, many facial traits capture variation that is largely shared across individuals, as they covary in predictable and structured ways. Recognizing this higher‐level organization allows trait‐based approaches to be interpreted within the framework of phenotypic integration, providing a more comprehensive understanding of how discrete traits are organized within individual faces and which trait combinations are likely or biologically constrained.

In this study, I examine how morphological modularity and integration shape the co‐occurrence of anatomical traits. Specifically, I evaluate alternative hypotheses of facial modularity and quantify patterns of integration between facial modules. Then, I assess how this integration pattern structures the joint occurrence of facial traits by comparing the observed prevalence of trait combinations with the prevalence expected under statistical independence.

## 2 MATERIALS AND METHODS

### 2.1 Modularity, integration, and continuous facial morphology

The dataset consists of 462 frontal facial photographs of adult males (mean age = 23.65; age range = 18–60) from the CARAMEX database, collected using a previously described standardized protocol (Serrano et al., 1999). Verbal informed consent was obtained from all participants photographed at the time of data collection, in accordance with the ethical standards and practices of the period. Only photographs with visible facial structures were included in the present study. Geometric morphometrics methods were used to evaluate the modularity and quantify the integration of facial variation. A total of 29 anatomical landmarks (LMs) were digitized on each image (Figure 1a), and LM configurations were standardized using Generalized Procrustes Analysis to remove variation associated with position, scale, and orientation (Zelditch et al., 2004).

**FIGURE 1:**
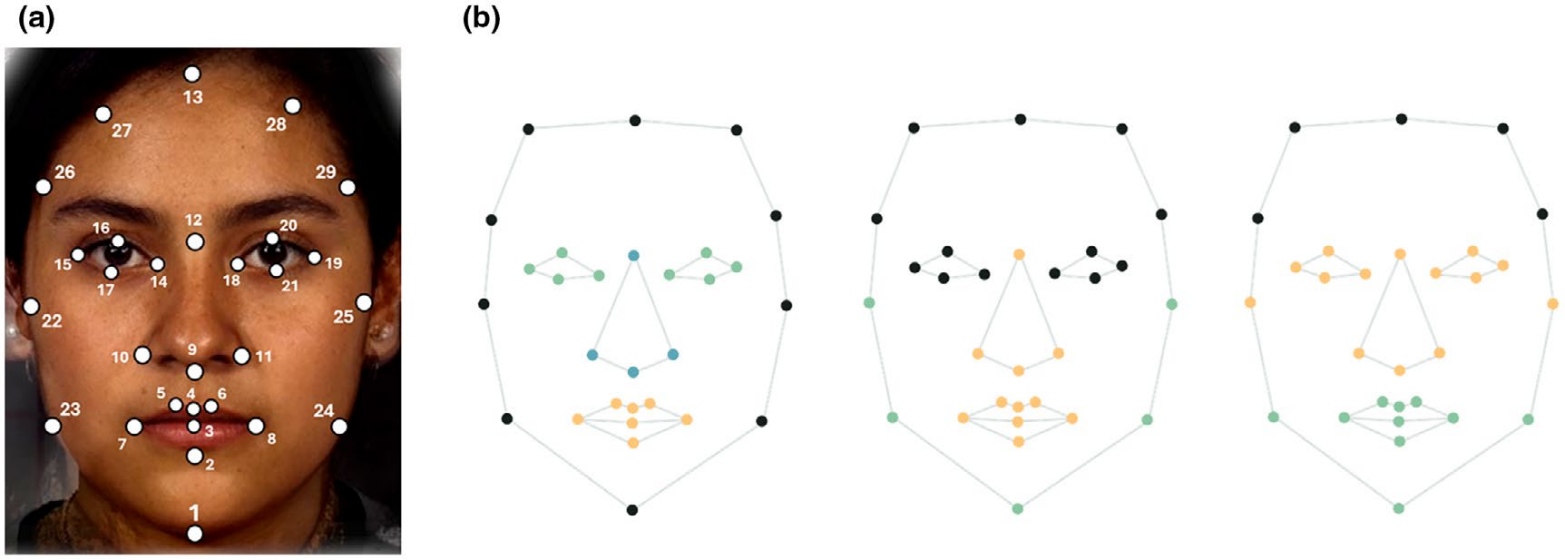
Landmark protocol and modularity hypotheses. (a) Landmark configurations showing face outline (LMs 1, 13, 22–29), eyes (LMs 14–21), nose (LMs 9–12), and mouth (LMs 2–8). The face image is synthetic and was generated using morphing of multiple facial photographs for illustrative purposes. (b) Modularity hypotheses evaluated from left to right: H1 Anatomical modules, H2 Developmental timing, H3 Facial thirds. Colors indicate module assignment under each hypothesis.

Procrustes coordinates were used to evaluate three alternative hypotheses of facial modularity (Figure 1b), each based on different biological or structural perspectives:

- H1 Anatomical modules. This hypothesis subdivides the face into four modules corresponding to major anatomical and functional units: eyes, nose, mouth, and facial outline. The facial outline includes landmarks on the forehead, photographic zygion, photographic gonion, and gnathion, representing the external skeletal contour visible in frontal view. This hypothesis is consistent with the functional matrix hypothesis (Moss & Young, 1960), which proposes that craniofacial morphology develops and varies in response to functional and structural relationships among soft tissues, cavities, and skeletal components.
- H2 Developmental timing. This hypothesis reflects differences in craniofacial growth trajectories and maturation timing. The forehead and eyes follow earlier growth trajectories, followed by midfacial structures (mouth and nose) and mandibular growth (Capote et al., 2023; Farkas et al., 1992).
- H3 Facial thirds. This hypothesis subdivides the face into three modules: the upper (forehead and orbits), middle (nose, zygion), and lower thirds (mouth, gonion, gnathion). This approach reflects common anatomical and clinical assessments of facial organization (Ciancia et al., 2024).

Modularity, defined as the relative independence among modules specified by each hypothesis, was evaluated using the covariance ratio (CR) (Adams & Collyer, 2019). The CR measures the covariance between two modules relative to within‐module covariances, with lower values indicating greater modularity (i.e., greater covariation within modules than between them). As all hypotheses include more than two modules, the average CR of all pairwise module comparisons is presented.

Statistical significance was assessed using 1000 permutation tests, in which the observed CR value was compared to a null distribution generated by randomly assigning landmarks to an equal number of modules as in the observed hypothesis. The strength of the modular signal among the hypotheses was compared using the standardized effect size (*Z*_CR_) (Adams & Collyer, 2019). The hypothesis with the lowest *Z*_CR_ was interpreted as providing the strongest support for the modular organization of the face, and subsequent analyses focused on the modules defined under this hypothesis.

Morphological integration, defined as the strength of the interdependency between the corresponding facial modules, was evaluated using two‐block partial least squares analysis (PLS). The statistical significance of integration was assessed using 1000 permutation tests, in which for each pair of modules, the observed PLS correlation (rPLS) was compared to a null distribution obtained by randomly assigning individuals between blocks (Bookstein et al., 2003). The strength of integration across pairs of modules was compared using the effect size of such correlation (*Z*_rPLS_), where zero indicates no integration and higher values indicate stronger integration.

Additionally, a principal component analysis (PCA) was performed on the Procrustes coordinates to characterize continuous patterns of facial covariation arising from modularity and integration across the whole face. Only principal components (PCs) explaining more than 10% of the total variance are reported.

### 2.2 Anatomical trait categorization

To examine whether covariation patterns observed in continuous facial traits are reflected in discrete phenotypic traits, morphometric variables (i.e., PC scores, centroid size, and linear distances) were transformed into categorical descriptions. Categories were defined in relation to the empirical population distribution using ±1 standard deviation (SD) thresholds. This approach, commonly used in anthropometric studies, captures the majority of the variation (68% of the population) within an intermediate category while allowing identification of relatively extreme phenotypic traits without treating them as statistical outliers.

Shape variation within each module was summarized using PCA. For a given anatomical region, the first PC (i.e., the main axis of shape variation) was selected, and categories were defined based on the observed shape pattern. Specifically, individual scores were categorized into three groups: values below and above 1 SD from the mean were interpreted as representing the extreme shapes along the corresponding shape continuum (e.g., angular vs. rounded forehead contour), while those within 1 SD were considered intermediate values.

Size information was quantified using centroid size, defined as the square root of the summed squared distance from each landmark in a module to the centroid. Additionally, linear dimensions were calculated as Euclidean distances between pairs of Procrustes coordinates to capture traditional relationships (e.g., eye separation). Both types of variables were categorized as small (below 1 SD), large (above 1 SD), and medium otherwise.

### 2.3 Joint prevalence of integrated discrete anatomical traits

The observed prevalence of trait combinations was calculated using categories within pairs of integrated facial modules. Expected joint prevalence under statistical independence was calculated as the product of the marginal frequencies of each trait category. Then, phenotypic dependency, defined here as the deviation from independence ratio (DIR), was quantified as the ratio between observed and expected joint prevalence. DIR values approximately equal to 1 indicate combinations consistent with statistical independence. Values greater than 1 indicate combinations occurring more frequently than expected. These patterns are consistent with redundancy arising from morphological integration, where the presence of one category increases the probability of observing others. Values less than 1 indicate combinations occurring less frequently than expected, potentially due to morphological constraints limiting certain joint occurrences.

To facilitate the interpretation of DIR values, two simulated datasets were generated under trait independence and trait dependence scenarios, each including the same number of individuals as the original dataset (*n* = 462). In the first scenario, trait categories were randomly assigned across individuals, producing combinations with no dependency structure, while in the second, trait categories were assigned to favor specific combinations, introducing covariation among traits.

All analyses were performed using R software (R Core Team, 2021) version 2024.12.1. Specifically, the CR values for each modularity hypothesis and the comparison of modularity effect sizes between hypotheses were calculated using the modularity.test and compare.CR functions in the geomorph package (Baken et al., 2021). PLS analyses and comparisons of integration strength between modules were calculated using the integration.test and compare.PLS functions within the same package.

## 3 RESULTS

### 3.1 Modularity, integration, and continuous facial variation

The modularity analysis showed support for all three hypotheses, with H1 showing the strongest modular signal (*Z*_CR_ = −3.91, *p* < 0.01), followed by H2 (*Z*_CR_ = −3.19, *p* < 0.01) and H3 (*Z*_CR_ = −2.58, *p* < 0.01). Based on these results, subsequent morphological integration analysis focused on the modules defined under H1.

The average integration among facial modules within the anatomical hypothesis (H1) was high (rPLS = 0.665, *Z*_rPLS_ = 11.12, *p* < 0.01). Pairwise *Z*_rPLS_ showed significant but varying degrees of integration across all module pairs (*p* < 0.01). The strongest covariation was observed between the facial outline and the eyes modules (Table 1).

**TABLE 1:** Integration between modules under the anatomical hypothesis (H1).

	Eyes	Mouth	Nose
Mouth	0.7149 (10.57)*		
Nose	0.4746 (6.58)*	0.5684 (8.72)*	
Outline	0.7922 (10.86)*	0.7858 (9.62)*	0.6515 (9.96)*

*Note*: Values represent rPLS coefficients, and effect sizes (*Z*_rPLS_) are shown in parentheses.

* *p* < 0.01.

Figure 2 shows the first three PCs accounting for 52.6% of total shape variance. PC 1 (21.3%) mainly captures variation in the width‐to‐height ratio of the face, while PC 2 (16. 8%) reflects variation in the hairline and the relative proportions of the eyes, nose, and mouth. PC 3 (14.5%) is mainly associated with variation in the facial outline.

**FIGURE 2:**
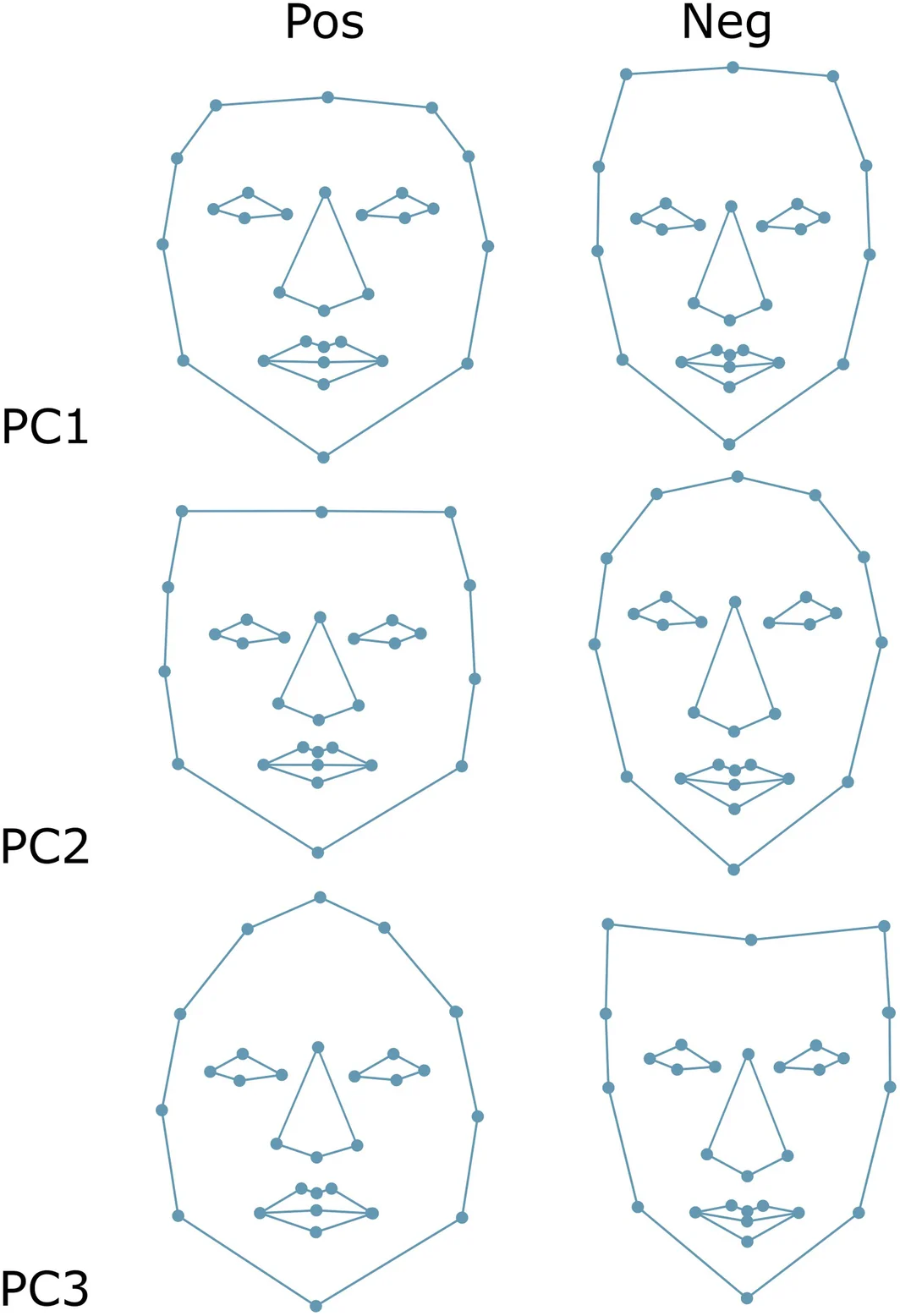
Visualization of shape variation along the first three principal components, showing the positive and negative extremes of facial morphology.

### 3.2 Anatomical trait categorization

Based on the best supported modularity hypothesis, eight facial traits were defined for the prevalence analyses by categorizing continuous morphometric variables (Table 2). The categories and modules shown in Table 2 correspond to those used in the prevalence analyses. For example, the outline–eyes module pair comprises face outline shape, eye tilt, eye size, and eye separation.

**TABLE 2:** Discrete facial traits derived from the anatomical modularity hypothesis (H1).

Facial trait	Module	Information source	Categories
Face outline shape	Face outline	First PC of facial contour	Wide and short, intermediate, tall and narrow
External eye canthus tilt	Eyes	First PC of eye shape	Upward, straight, downward
Eye size	Eyes	Mean centroid size of the left and right eye configurations	Small, medium, large
Eye separation	Eyes	Distance between the inner eye corners	Close, intermediate, wide
Nose width proportion	Nose	Ratio between nasal height (nasion‐subnasale) and nasal width (alare‐alare)	Wide base, proportioned, narrow base
Nose size	Nose	Centroid size of the nose configuration	Small, medium, large
Lip shape	Mouth	First PC of the mouth configuration	Full, intermediate, thin lips
Mouth size	Mouth	Centroid size of the mouth configuration	Small, medium, large

### 3.3 Joint prevalence of integrated discrete anatomical traits

Figure 3 shows the distribution of DIR values for the observed and simulated datasets. Overall, the observed data show a broader distribution, with a higher frequency of low and high values across module pairs (outline–eyes: q25 = 0.742, q50 = 1.33, q75 = 2.97, SD = 0.466; outline–mouth: q25 = 0.779, q50 = 0.977, q75 = 1.51, SD = 0.292; outline–nose: q25 = 0.315, q50 = 0.742, q75 = 1.45, SD = 0.526; eyes–mouth: q25 = 0.871, q50 = 1.41, q75 = 3.63, SD = 0.470; Eyes – Nose: q25 = 0.799, q50 = 1.46, q75 = 3.84, SD = 0.463; nose–mouth: q25 = 0.569, q50 = 1.02, q75 = 2.25, SD = 0.435). In contrast, the independent dataset shows a narrower distribution centered around 1 (q25 = 0.741, q50 = 0.989, q75 = 1.23, SD = 0.185), and the dependent dataset is shifted toward higher values (q25 = 2.54, q50 = 2.89, q75 = 3.41, SD = 0.107).

**FIGURE 3:**
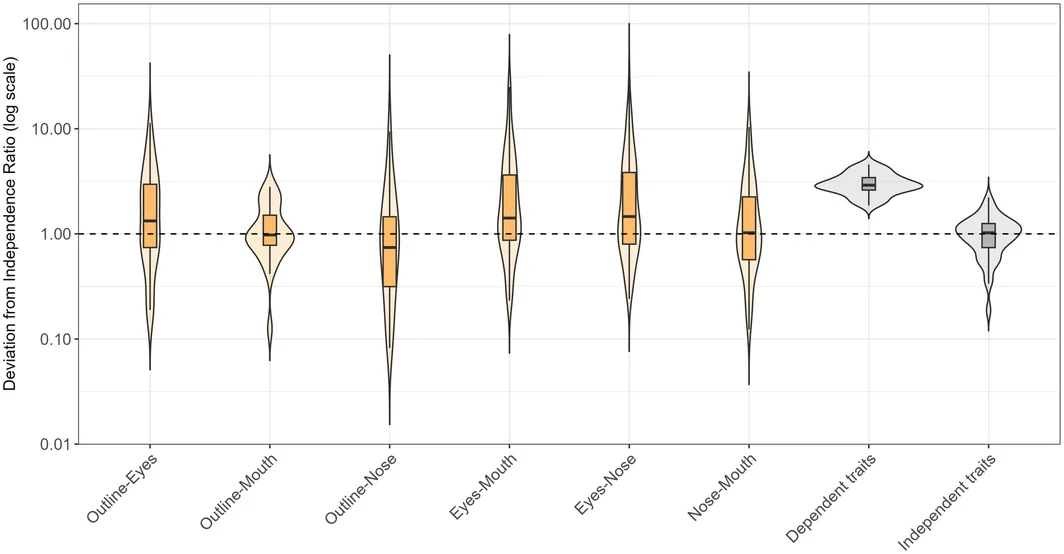
Distributions of the deviation from independence ratio (DIR) on a logarithmic scale for observed trait combinations derived from module pairs under the anatomical modularity hypothesis (H1). Simulated distributions under independence and dependence scenarios are shown for comparison. Violin plots illustrate the full distribution density, and the box plots indicate the median and interquartile range.

These results indicate that the observed joint prevalence of trait combinations deviates from both complete independence and strong dependence. Ratios greater and lower than 1 suggest structured dependencies among facial traits rather than random co‐occurrence and are consistent with the patterns identified in the morphological integration analysis of continuous data.

Some examples of trait combinations occurring less frequently than expected under independence (DIR values below an arbitrary threshold of 0.5) for each module pair include:

- Outline–eyes: intermediate face outline with straight eye tilt, small eye size, and wide eye separation; tall and narrow face outline with straight eye tilt, intermediate eye size, and close eye separation.
- Mouth–eyes: intermediate mouth shape and mouth size, with downward eye tilt, intermediate eye size, and intermediate eye separation; thin lips with intermediate mouth size, downward eye tilt, intermediate eye size, and intermediate eye separation.
- Nose–eyes: large nose with a narrow base, straight eye tilt, intermediate eye size, and eye separation; medium‐sized nose with a wide base, upward eye tilt, intermediate eye size, and intermediate eye separation.
- Nose–mouth: medium‐sized nose with a narrow base, full lips, and intermediate mouth size; intermediate‐sized nose with a wide base, thin lips, and intermediate mouth size.
- Outline–mouth: tall and narrow face outline with intermediate lip shape and large mouth size; wide and short face outline with intermediate lip shape and small mouth size.
- Outline–nose: tall and narrow face outline and a large nose with an intermediate base; tall and narrow face outline and a small nose with an intermediate base.

## 4 DISCUSSION

This study examined how both continuous and discrete facial traits covary and co‐occur within a population of adult males. These approaches converge in supporting the structured organization of facial variation. In particular, analyses on continuous variation indicate that facial shape is organized into semi‐autonomous units, with the strongest support for modules corresponding to the Anatomical modularity hypothesis (face outline, eyes, nose, mouth). At the same time, the results showed varying levels of integration between anatomical modules, with the highest integration observed between the facial outline and eyes modules and between the mouth and eyes modules, whereas module pairs involving the nose showed moderate integration. Additionally, PCA of facial shape provided a complementary summary of covariation patterns, showing that the main axes of covariation were primarily associated with the width‐to‐height ratio and shape of the facial outline, as well as with the relative proportions of the eyes, nose, and mouth.

These findings align with previous studies describing structured covariation patterns in facial soft tissues in other populations, reflecting underlying developmental, functional, and social processes (Esteve‐Altava et al., 2015; Quinto‐Sánchez et al., 2018; Sheehan & Nachman, 2014; Starbuck et al., 2011). Particularly, the integration patterns observed here are consistent with evidence of coordinated growth between orbits and adjacent skeletal morphology (Liang et al., 2023; Moss & Young, 1960), as well as with shared growth patterns between nasal and oral cavities and their relationships with the maxillary and subnasal regions, and facial height (Landi et al., 2022).

The observed interdependence among facial modules is also compatible with psychological research on face perception, which emphasizes that while single features can be processed independently (featural processing), facial features and their spatial relationships are largely processed holistically (Boutet et al., 2021; Cabeza & Kato, 2000; Piepers & Robbins, 2012). Eye size, for example, is perceived relative to the facial context rather than in isolation (Xiao et al., 2014). This perceptual evidence is also consistent with the strong phenotypic integration observed between the facial outline and eyes modules. Although speculative, these results suggest that the coordinated anatomical variation of the face may contribute to the perceptual integration of facial features, with potential implications for improving training strategies for examining facial variation. However, further research in this area is needed.

The prevalence analyses of observed and simulated discrete facial traits likewise showed that facial trait combinations do not occur randomly but reflect interdependencies predicted by modularity and integration, likely arising from shared biological processes or constraints. Some module pairs showed distributions close to independence (median ≈ 1), while others showed deviations above or below 1, indicating likely or constrained combinations of traits. Such heterogeneity in DIR distributions suggests that morphological integration, as captured through discrete traits, is not uniform, which is consistent with the results on continuous variation presented above.

Compared to previous studies that mainly focused on describing the statistical prevalence of individual traits or trait combinations (Rani et al., 2022; Ritz‐Timme et al., 2011; Roelofse et al., 2008), the present study offers a biological explanation for such patterns. Additionally, these results encourage us to reconsider how individualizing characteristics are conceptualized. While rare traits (e.g., nevi non‐cranial traits) remain useful for distinguishing individuals, combinations of more common traits can also contribute to individualization when their co‐occurrence is biologically constrained and, thus, relatively uncommon within the population.

Overall, the present study demonstrates the utility of concepts of phenotypic modularity and integration as theoretical and analytical frameworks for interpreting discrete facial traits as components of integrated systems, complementing traditional trait‐based approaches. While current practices in the fields of forensic identification, clinical dysmorphology, and biological anthropology often describe and record traits individually, adopting a higher‐order perspective means recognizing that isolated traits capture only a subset of the meaningful variation present in facial phenotypes. This perspective can inform more theoretically grounded strategies for documenting and interpreting the biological significance of subsets of facial discrete features.

## AUTHOR CONTRIBUTIONS

Arodi Farrera: conceptualization, acquisition of data, data analysis and interpretation, drafting of the manuscript.

## Data Availability

The data that support the findings of this study are available on request from the corresponding author. The data are not publicly available due to privacy or ethical restrictions.
